# Supplementary material for: Facility Managers’ Perceptions of Support and Supervision of Ward Based Primary Health Care Outreach Teams in National Health Insurance Pilot Districts in KwaZulu-Natal, South Africa. A Qualitative Study
Source: Healthcare (Basel). 2021 Dec 13;9(12):1718. doi: 10.3390/healthcare9121718 (PMC8701860; doi:10.3390/healthcare9121718)
Supplement: Supplementary file 1 [file healthcare-09-01718-s001.zip › healthcare-1352308-supplementary.pdf]

healthcare MDPI

Supplementary Material: Ward Based PHC Outreach Facility Manager Interview

Participant Code:

Date of Interview:

A. Characteristics of ward based Outreach Facility Manager

1. Tell us about yourself

Probes:

1.1. Age

1.2. Gender

1.3. What is your highest educational qualification?

1.4. Do have PHC training after qualifying as a nurse

2. Tell me more about your current job

Probes

2.1. What is your current job title?

2.2. For how long have you been in this position in this facility?

3. Tell me when did you start working as a Facility Manager?

Probes

3.1. Explain if there is any training you received to prepare for the implementation of ward based PHC outreach teams

3.1. Tell me more about the training you had.

Do you need more training? In which areas and why?

B. Support and Supervision

4. What do you understand to be your role as a Facility Manager in the implementation of ward based PHC outreach teams?

5. Tell me how do you provide support and supervision to the outreach team?

Probes

5.1 Do you meet with team leader to discuss her work and team progress?

5.2 Do you meet with CHWs in the team to discuss their work and progress?

5.3 Do you accompany the CHWs to the households?

5.4 Do you ensure they have the equipment/transport they need?

6. Are you aware of the challenges experienced by the teams providing services in communities

Probes

6.1 Give some examples of these challenges

6.2. Tell me how have you addressed these challenges?
6.3. What challenges do you yourself face in providing support and supervision to the teams? How have you addressed these challenges? If you have been unable to address them, please explain the factors impeding progress?

7. Are there any successes worth sharing regarding teams' experiences?
7.1 Give some examples

8. Do you think the WBOTS are effective in improving health and access to health care in KZN? Please explain your answer

C.REFERRAL LINKAGES TO CLINIC

9. Explain how CHWs refer clients to the clinic?

10. Explain how clinic staff manages these referrals?

11. Explain how clinic staff provide feedback to the team leader?
What are the challenges encountered with referrals (by staff and patients)?

12. Tell me how you address challenges you face with referrals from CHWs?

13. Explain how clinic staff provides back referrals to CHWs?

14. Explain if the upward and downward referral add any value
Probe
12.1. Give examples

D.RESOURCES

15. Tell me about what happens when the outreach teams do not have supplies they need?
16. Explain where you get the supplies from.
17. What role does the clinic play in providing supplies to the outreach teams
18. Does the clinic provide meeting space for the teams?
Probe
18.1 Where do they meet?
19. How do CHWs access the transport they need to get to households? Are there any challenges with this process? How have you tried to address these? Have you been successful? If not, why not?

Thank you for taking time to participate in this interview

(Adapted from Whyte, 2015)
